# Gene co-expression networks reveal sex-biased differences in musculoskeletal ageing

**DOI:** 10.3389/fragi.2024.1469479

**Published:** 2024-09-18

**Authors:** Samael Olascoaga, Hugo Tovar, Jesús Espinal-Enríquez

**Affiliations:** ^1^ Posgrado en Biología Experimental, División de Ciencias Biológicas y de la Salud, Universidad Autónoma Metropolitana Iztapalapa, Mexico City, Mexico; ^2^ Computational Genomics Division, National Institute of Genomic Medicine, Mexico City, Mexico

**Keywords:** gene co-expression networks for ageing, functional enrichment analysis, loss of function in ageing, sexual dimorphism in ageing, loss of gene co-expression in ageing, musculoskeletal ageing

## Abstract

Aging is a universal and progressive process involving the deterioration of physiological functions and the accumulation of cellular damage. Gene regulation programs influence how phenotypes respond to environmental and intrinsic changes during aging. Although several factors, including sex, are known to impact this process, the underlying mechanisms remain incompletely understood. Here, we investigate the functional organization patterns of skeletal muscle genes across different sexes and ages using gene co-expression networks (GCNs) to explore their influence on aging. We constructed GCNs for three different age groups for male and female samples, analyzed topological similarities and differences, inferred significant associated processes for each network, and constructed null models to provide statistically robust results. We found that each network is topologically and functionally distinct, with young women having the most associated processes, likely due to reproductive tasks. The functional organization and modularity of genes decline with age, starting from middle age, potentially leading to age-related deterioration. Women maintain better gene functional organization throughout life compared to men, especially in processes like macroautophagy and sarcomere organization. The study suggests that the loss of gene co-expression could be a universal aging marker. This research offers insights into how gene organization changes with age and sex, providing a complementary method to analyze aging.

## Introduction

The medical and technological progress of contemporary societies has led to a notable increase in the average life expectancy of humanity. As a result, the aging population is experiencing constant and rapid growth. Consequently, there is a significant increase in the incidence of chronic diseases. This phenomenon is largely due to the decisive role that chronological age plays as a predisposing factor for conditions such as diabetes (Longo et al., 2019), cardiovascular diseases (North and Sinclair, 2012), respiratory diseases (Cho and Stout-Delgado, 2020), cancer (Berben et al., 2021), and musculoskeletal problems (Briggs et al., 2016).

Aging is a universal and progressive process of deterioration of physiological functions and the accumulation of damage in cells and tissues, ultimately leading to death (Guo et al., 2022). Although the processes that trigger and drive aging are not yet fully understood, a series of 12 hallmarks that drive and accelerate this process have been proposed. These include genomic instability, telomere shortening, epigenetic alterations, loss of proteostasis, deactivation of autophagy, deregulation in nutrient sensing, mitochondrial dysfunction, cellular senescence, loss of stem cells, alterations in intercellular communication, chronic inflammation, and imbalance of microbial homeostasis (dysbiosis) (López-Otín et al., 2023).

The biological and molecular processes that drive life are complex and highly regulated phenomena, controlled primarily by the functional organization of genes rather than their expression levels. It has been suggested that gene organization and expression are structured in modular networks (Mark et al., 2019). However, it is known that this functional organization and modularity tend to diminish or be lost with age (Southworth et al., 2009a), which could lead to malfunctioning cellular processes. The causes of this loss of functional coordination due to aging are currently unknown. Nonetheless, it has been proposed that aging has a stochastic component, related to the accumulation of regulatory errors in transcription, translation, and metabolism pathways due to the inefficiency of signaling cascades in the network and responses to environmental factors (Podolskiy et al., 2015; Lea et al., 2019). Other unknown aspects of the loss and decline in functional organization include the life stage at which it begins and the influence of sex.

Functional organization within the cell or an organism is not solely determined by gene expression levels but is rather the result of a delicate process of co-regulation between genes, transcription factors, and non-coding species (Sinha et al., 2020; Yap and Greenberg, 2018). This co-expression regulation process more accurately reflects the overall state of a cell over time (Glass et al., 2013). Frequently, approaches from physics and computational sciences have been employed to explore the co-expression patterns within a phenotype. A prime example of such an approach is co-expression networks (Mähler et al., 2017; Hernández-Lemus et al., 2019). This tool can help observe globally how pairs of genes maintain dependent relationships within a given phenotype (Ovens et al., 2021; García-Cortés et al., 2020). These relationships, collectively, can reflect the state of a phenotype and, at the same time, serve to compare different phenotypes, either between conditions or the same condition at different points in time (Espinal-Enríquez et al., 2017; Nakamura-García and Espinal-Enríquez, 2023).

In this study, through the analysis of gene co-expression networks, we explore the changes that occur in the transcriptome structure across three life stages: youth, midlife, and aging. Additionally, we analyze the differences in the functional organization of the transcriptome between men and women, using skeletal muscle as our study model, which is a postmitotic tissue. Postmitotic cells are an excellent system for studying the effects of aging, as they originate from mitotic stem cells that irreversibly lose their ability to proliferate upon differentiation. These cells can persist and function for extended periods within the organism, accumulating significant damage and aberrations that impact their function. These cells cannot be stimulated to proliferate by any physiological stimulus, nor by non-physiological stimuli such as oncogene expression or radiation. Unlike mitotic cells, postmitotic cells never undergo tumorigenic transformation (Campisi and Warner., 2001).

## Methods

### Data acquisition and pre-processing

**FIGURE 1:**
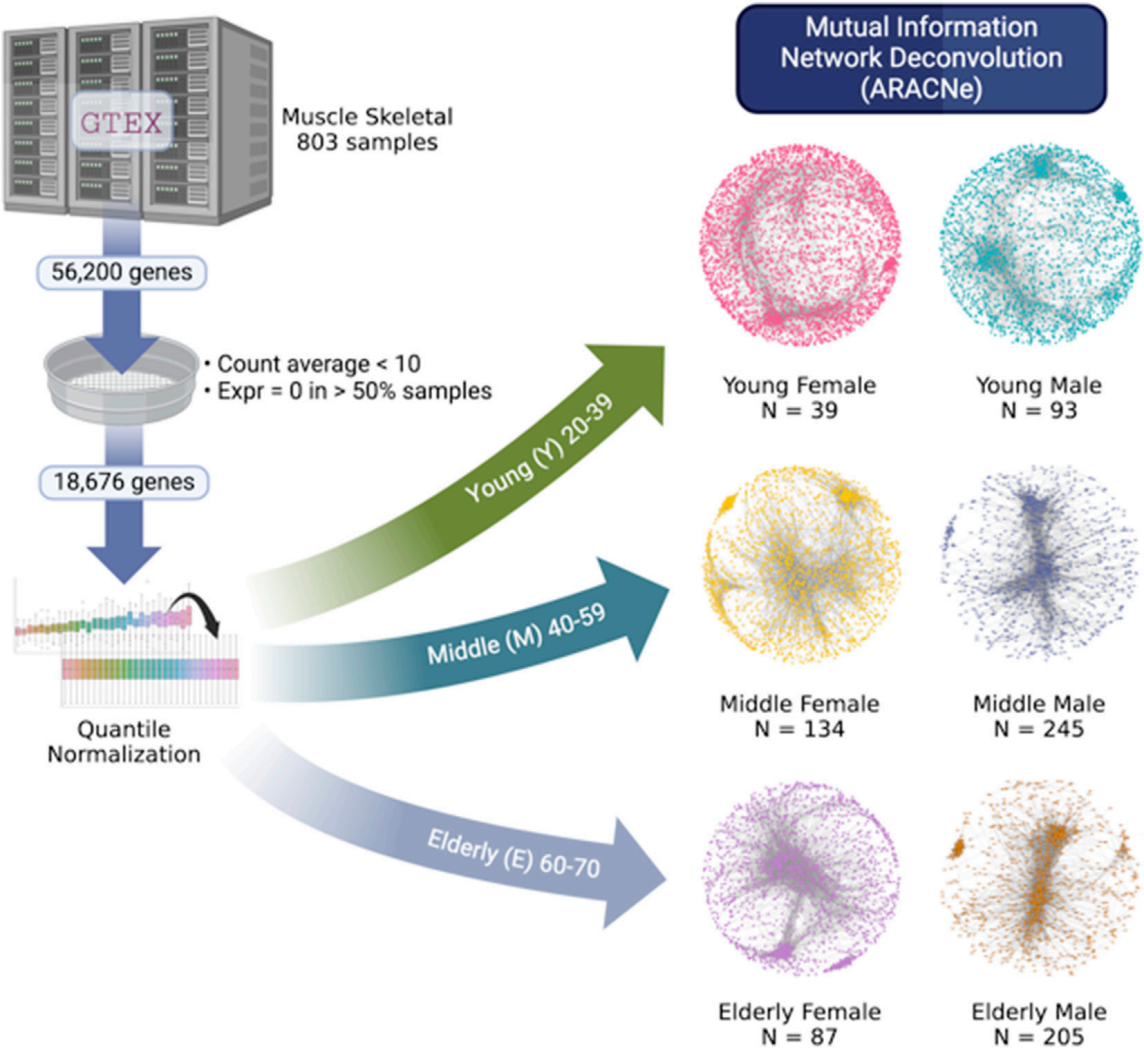
Graphical representation of the workflow presented here.

Figure 1 is a graphical representation of the workflow implemented in this work. Gene expression data (raw counts) were obtained through RNA-seq from the GTEx Analysis V8 project (GTEx Consortium, 2020). Classically, biological properties have been compared between young and elderly individuals, almost always finding very marked differences. However, these differences could have arisen in stages prior to aging, such as middle age, as various authors have reported that the first signs of multiple diseases begin during this stage (Taylor et al., 2019; Dmitrieva et al., 2023; Blümel et al., 2020). Therefore, we also selected tissue samples from mature individuals. We selected 803 samples of healthy human skeletal muscle, free of any pathology. The data were divided according to sex and three different age ranges: Young (20–39 years, N: F = 39, M = 93), Middle-aged (40–59 years, N: F = 134, M = 245), and Elderly (60–70 years, N: F = 87, M = 205), as defined by the GTEx project.

A dataset was generated with a total of 803 samples (columns), each with a total of 56,200 genes (rows). The mean counts for each gene across the entire dataset were calculated, and genes with a mean less than 10 were removed. Additionally, genes with zero counts in more than 50% of the samples were also eliminated (García-Cortés et al., 2022). After this processing, 18,676 genes remained, including protein-coding genes, long non-coding RNAs, microRNAs, pseudogenes, and other RNA species. The entire dataset was then normalized by quantiles using the qnorm 0.8.1 package. The next step involved separating the dataset according to the three age ranges: Young (20–39 years), Middle-aged (40–59 years), and Elderly (60–70 years). Within each age range, the data were divided into males and females, resulting in six distinct datasets. This entire process was conducted using in-house Python scripts.

### Gene co-expression network (GCN) inference

To study the functional coordination of genes, we utilized Gene Co-Expression Networks (GCNs), inferred using mutual information (MI) as a measure of gene co-expression. ARACNe is one of the most popular methods for GCN inference; the algorithm calculates the MI between two data series (Margolin et al., 2006). We applied ARACNe to each of the six datasets to establish correlations between pairs of genes. To accelerate the calculation, we used the multi-core C++ version without adaptive partitioning inference (Andonegui-Elguera et al., 2021), available at: https://github.com/josemaz/aracne-multicore. We decided to analyze and retain the 10,000 strongest interactions for each network to ensure the same size for the six networks, making them comparable. The choice of network size has been reported in various studies (Andonegui-Elguera et al., 2021; Velazquez-Caldelas et al., 2019; Zamora-Fuentes et al., 2020) as significant for analyzing structural and functional characteristics. Finally, network visualization was performed using Cytoscape v3.9.1.

### Analysis of interactions and shared genes

Shared interactions between the six different networks were identified. We used an algorithm that creates a dictionary of interactions using frozensets to ensure no duplication of interactions. Then, an empty matrix was created using the interaction dictionary as indices and columns. The interaction sets in the dictionary were traversed, and shared interactions between each pair of sets were calculated, filling the matrix with the corresponding values. Finally, the matrix elements were converted to integers, and a Boolean mask was created for the lower triangle and the diagonal of the matrix. Seaborn and Matplotlib were used to create a heatmap of the upper triangle of the intersection matrix.

To compare the shared genes between pairs of networks within the top 10,000 strongest interactions of each network, the algorithm followed the same approach.

Unique interactions in each network were also analyzed and identified. Briefly, the algorithm created a dictionary to store the interactions of each network, with each interaction represented as an alphabetically ordered tuple of involved genes to avoid duplications. An iterative process was used to compare the interaction sets of each network and find interactions unique to a particular network.

### Functional enrichment and statistical significance

The genes comprising the different networks were enriched within the Gene Ontology categories 2021 to observe the biological processes they belong to. For this purpose, we used Enrichr (https://maayanlab.cloud/Enrichr/) through its GSEApy API (Fang et al., 2023), with statistical significance determined by an adjusted $P_{\mathrm{val}}<0.05$ . The overlap of biological functions between the different networks was calculated using the from_memberships function of the Upsetplot 0.8 library, and the visualization was constructed with the plot function of the same library.

To determine if the structure of GCNs erodes due to aging, we used bootstrapping. We quantified unique interactions for each sex in each age range and then generated a null model consisting of randomly selecting samples from the original 10,000 connections. Each sample had the same size as the original network, and 100,000 simulations were performed per network. The probability was calculated by counting the number of times the original size resulted in a random network and dividing that number by the number of simulations.

## Results

### Changes in the structure of muscle GCNs over the years

**FIGURE 2:**
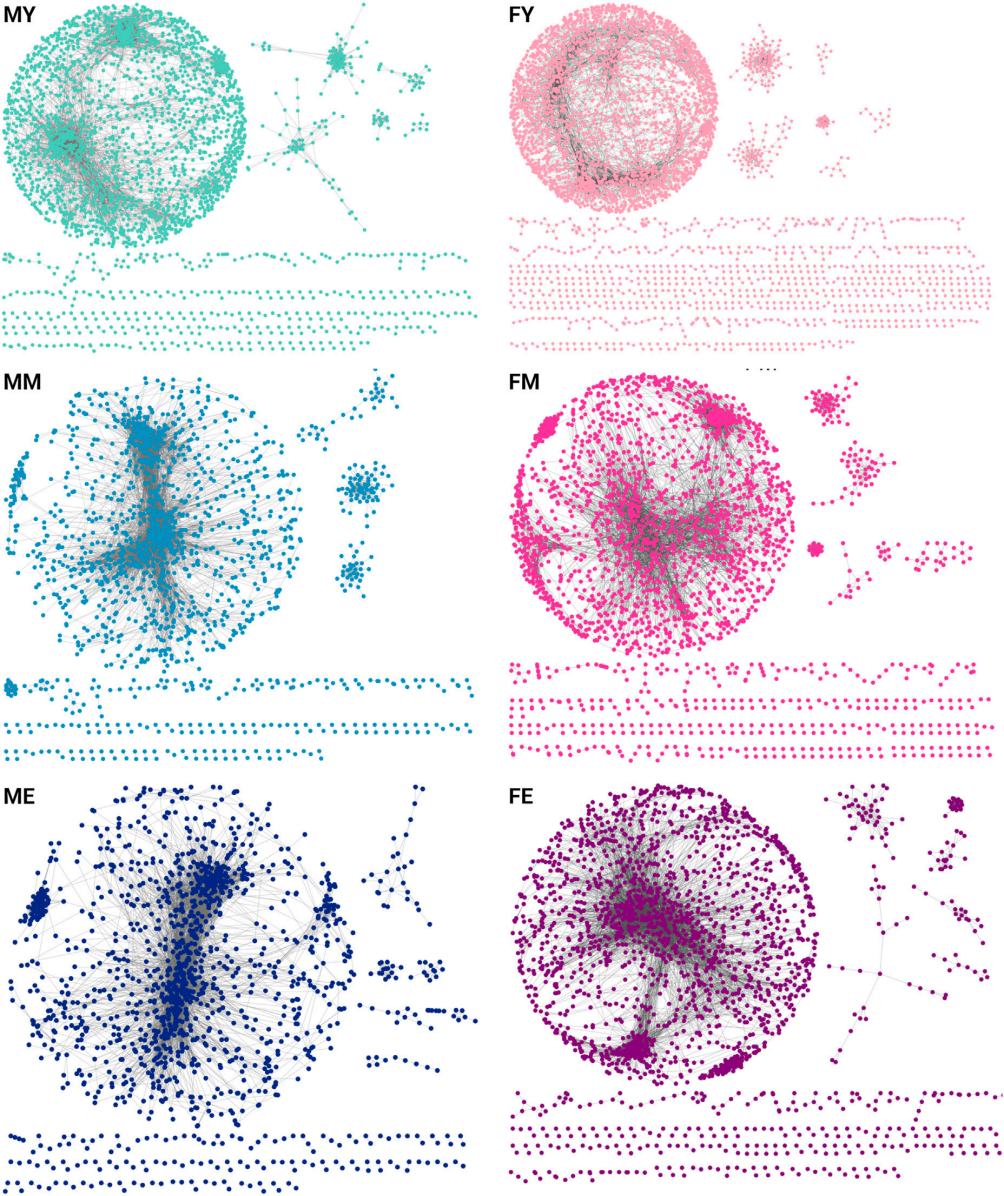
GCNs for each age group separated by sex [(F)emale and (M)ale]. Each network contains the top-10,000 gene-gene interactions, extracted for each network of young (Y), middle-aged (M), and elderly (E) age groups.

To understand how transcriptomic changes in skeletal muscle develop and vary according to sex and age, we analyzed expression profiles in men and women of different ages. We constructed and analyzed GCNs using mutual information (MI) as a measure of association. We calculated the co-expression of the entire transcriptome, and to make the networks comparable, we selected the top 10,000 strongest interaction pairs for each network. Figure 2 shows the GCNs generated for men (M) and women (F) in young (Y), middle-aged (M), and elderly (E) age groups (Supplementary Material S1 contains the edgelists for each phenotype). All six networks exhibit a highly connected giant component and several smaller components. This network structure suggests a well-defined and highly conserved transcriptional program throughout different life stages. However, a slight loss of density in the networks is also observed over the years. In Supplementary Material S2 the genes that are loss in the transition from young to middle ages in males and females are shown.

**FIGURE 3:**
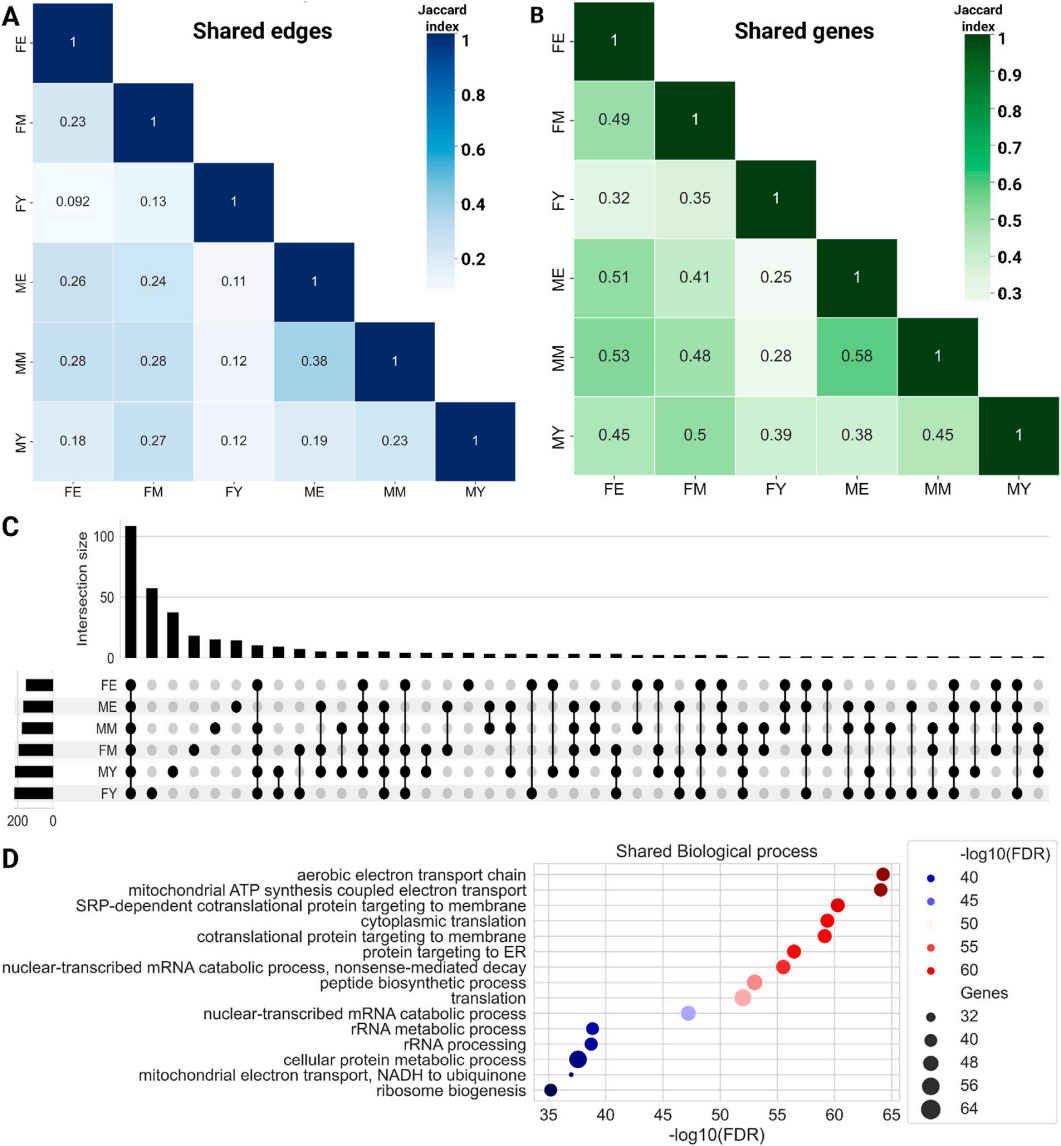
Heatmaps for shared edges **(A)** and gene **(B)** interactions among the six networks. Color intensity is proportional to the amount of shared elements between groups. **(C)** Upset plot showing the biological processes shared among the complete networks. **(D)** Common biological processes for all phenotypes.

To evaluate whether this transcriptional program is conserved across different life stages, we quantified the number of shared links (Figure 3A) and genes (Figure 3B) among the six networks. We show the similarity measured by the Jaccard index of shared links, i.e., the same genes connected by the same edges. The MM and ME networks share the most interactions, suggesting that the functional coordination of genes remains relatively stable during mid and late life stages, without abrupt changes in the transition from middle age to old age. The most notable changes seem to occur during the transition from youth to middle age, with a loss of connections becoming more pronounced during aging. This trend appears to be consistent among women, as the FM and FE networks share more interactions than FY with FM and much more than FY with FE.

Additionally, we identified the genes shared among the networks, as shown in bottom panel of Figure 3B. To note, the trend observed with shared links is maintained with shared genes. ME and MM share the highest proportion of genes, while the young groups share a lower proportion of genes with both older groups. Similarly, FE and FM share a higher proportion of genes than young women with the other two groups. Together, these results suggest that network connectivity and the genes that comprise them are lost abruptly during the transition from youth to middle age. However, this trend of losing connections and genes does not continue or worsen during old age.

Since the largest component of the six networks contains the majority of genes, we also calculated the shared genes in the giant component for the three ages in female and male networks. The results are presented as Venn diagrams in Supplementary Material S3. As it can be observed in the figures, two main results appear from those figures, the number of shared genes is high in both cases, and also, for young phenotypes, there are several unique genes in the giant component.

### Functional enrichment analysis

To compare the biological processes among the different networks, we performed an enrichment analysis of ontological terms. Figure 3C shows the biological processes enriched with an FDR<0.05 shared among the complete networks. All six networks have 108 common biological processes. Figure 3D shows the top 15 most enriched biological processes shared by all six networks, primarily finding metabolic and bioenergetic processes such as the electron transport chain, mitochondrial ATP production, protein synthesis and processing, RNA catabolism, and ribosomal biogenesis. This suggests that the functional organization of the genes responsible for these processes is maintained over the years, possibly indicating that these processes are crucial for the proper functioning and maintenance of skeletal muscle.

On the other hand, each network also has unique biological processes not shared with any other network, with FY having the highest number of unique processes (57), followed by FM (18), and finally FE, which has the fewest unique biological processes (4): cellular hyperosmotic response, internal protein amino acid acetylation, tail-anchored membrane protein insertion into ER membrane, and peptidyl-lysine methylation.

This trend of losing unique processes changes slightly in men, with MY having 37 unique processes, MM 15, and ME 14. It is interesting to note that in both women and men, the loss of biological processes occurs abruptly in middle age, which is consistent with the loss of connections and genes in the GCNs. Supplementary Material S2 shows the biological processes that are loss in the transition. The total unique biological processes for each network and those shared among different group combinations can be found in Supplementary Material S4.

### Age- and sex-specific transcriptional programs

**FIGURE 4:**
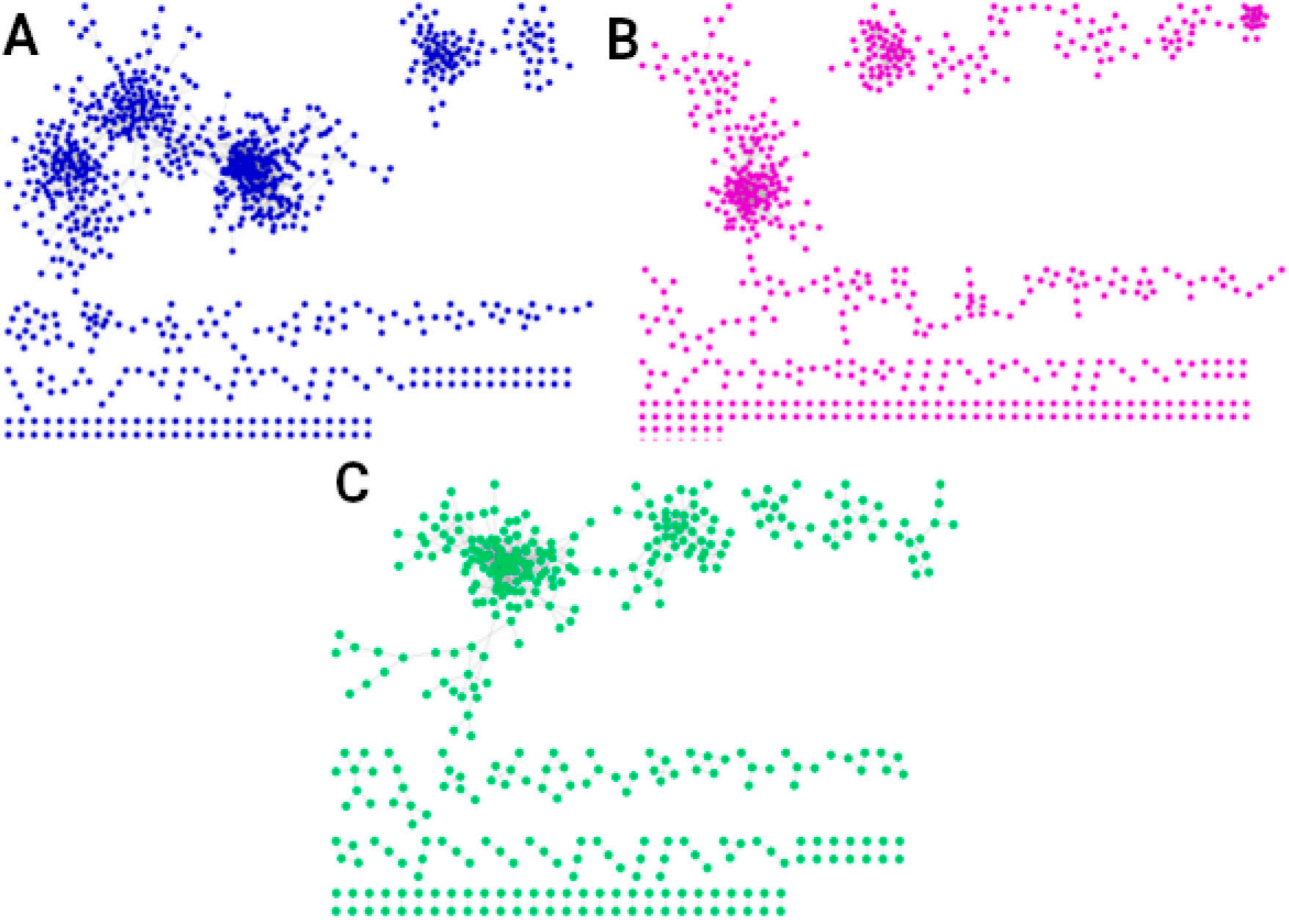
Networks of the shared interactions (same genes, same links) between **(A)** men of all three ages (2,557 interactions), **(B)** women of all ages (1,350 interactions), and **(C)** men and women of all ages (859 interactions). The interactions shared by men and women in all three age groups, shown in **(C)**, suggest the existence of a universal transcriptional program that could regulate essential universal processes to maintain the organism alive and functional.

We also quantified the interactions shared exclusively by men in the three age groups, identifying 2,557 conserved interactions (Figure 4A). These interactions enrich only one biological process: positive regulation of RNA metabolic process. For women in all three age groups, 1,350 interactions are conserved (Figure 4B), associated with six biological processes: macroautophagy, sarcomere organization, vesicle coating, protein ubiquitination, vesicle targeting, rough ER to cis-Golgi and COPII vesicle coating. The interactions shared by men and women of all ages are 859 (Figure 4C), with these shared interactions mainly involved in mitochondrial biogenesis and assembly, translation and RNA processing, respiration, and energy production.

**FIGURE 5:**
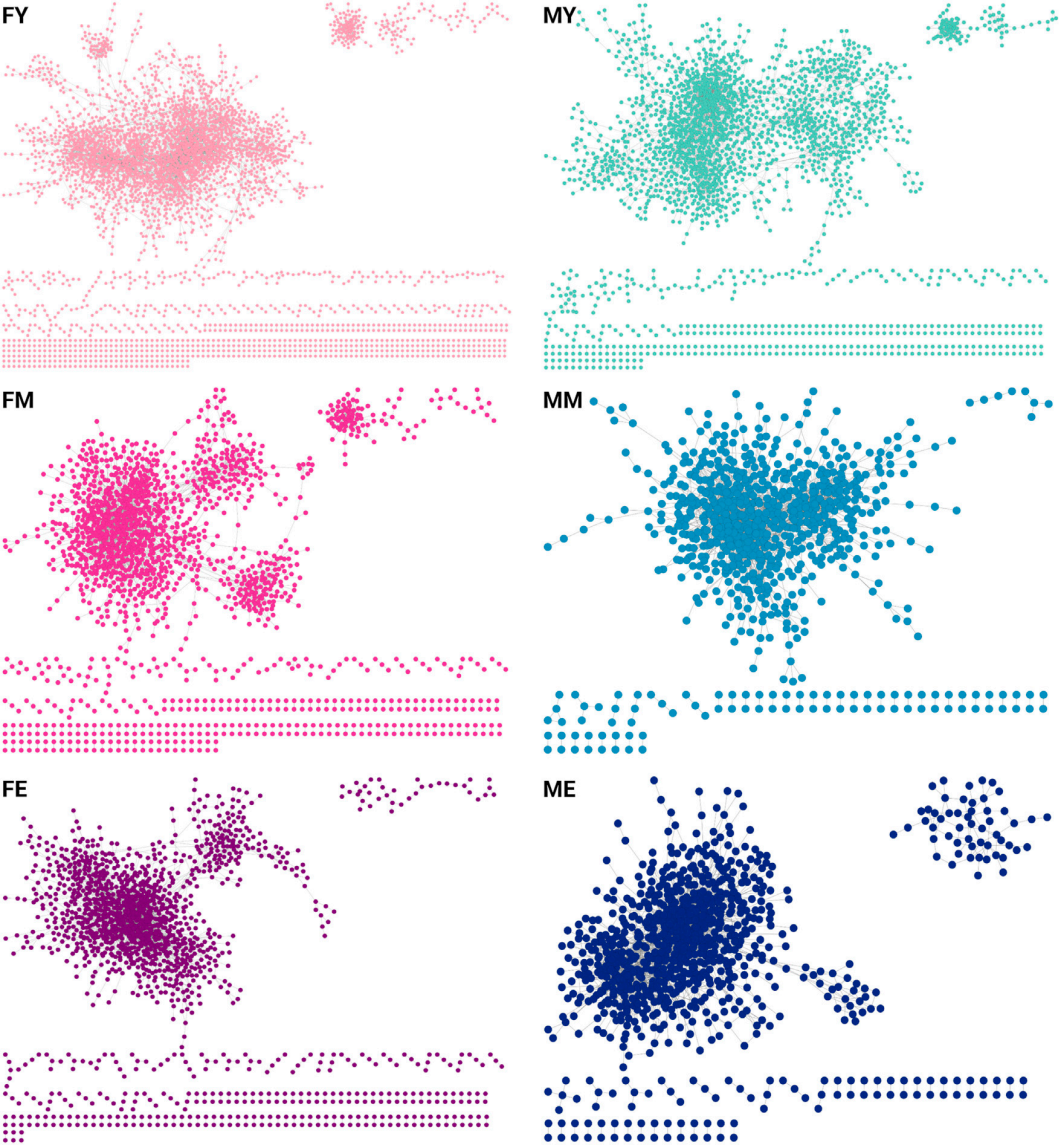
Unique interactions of each network. The women’s network with the highest number of unique interactions is the young (6,605), followed by the elderly (3,733), and finally middle-aged women (3,047). For men, the network with the highest number of unique interactions is the young (4,054), followed by the elderly (2,745), and finally middle-aged men with the fewest unique interactions (2,180).

We also obtained the unique interactions for each network, i.e., the unique and different transcriptional programs for each age and sex, as shown in Figure 5. Table 1 summarizes the number of links and nodes that comprise the unique interaction networks, as well as the size of the largest group of interconnected genes. The women’s network with the highest number of unique interactions is FY (6,605), followed by FE (3,733), and finally FM (3,047). For men, the network with the highest number of unique interactions is MY (4,054), followed by ME (2,745), and finally MM with the fewest unique interactions (2,180).

**TABLE 1:** Topological features that comprise the unique interaction networks.

Group	Edges	Total genes	LCC
FY	6,605	3,723	2,628
FM	3,047	1,671	1,194
FE	3,733	1,607	1,246
MY	4,054	2,283	1,738
MM	2,180	800	706
ME	2,745	902	758

*LCC*, Largest connected component.

**FIGURE 6:**
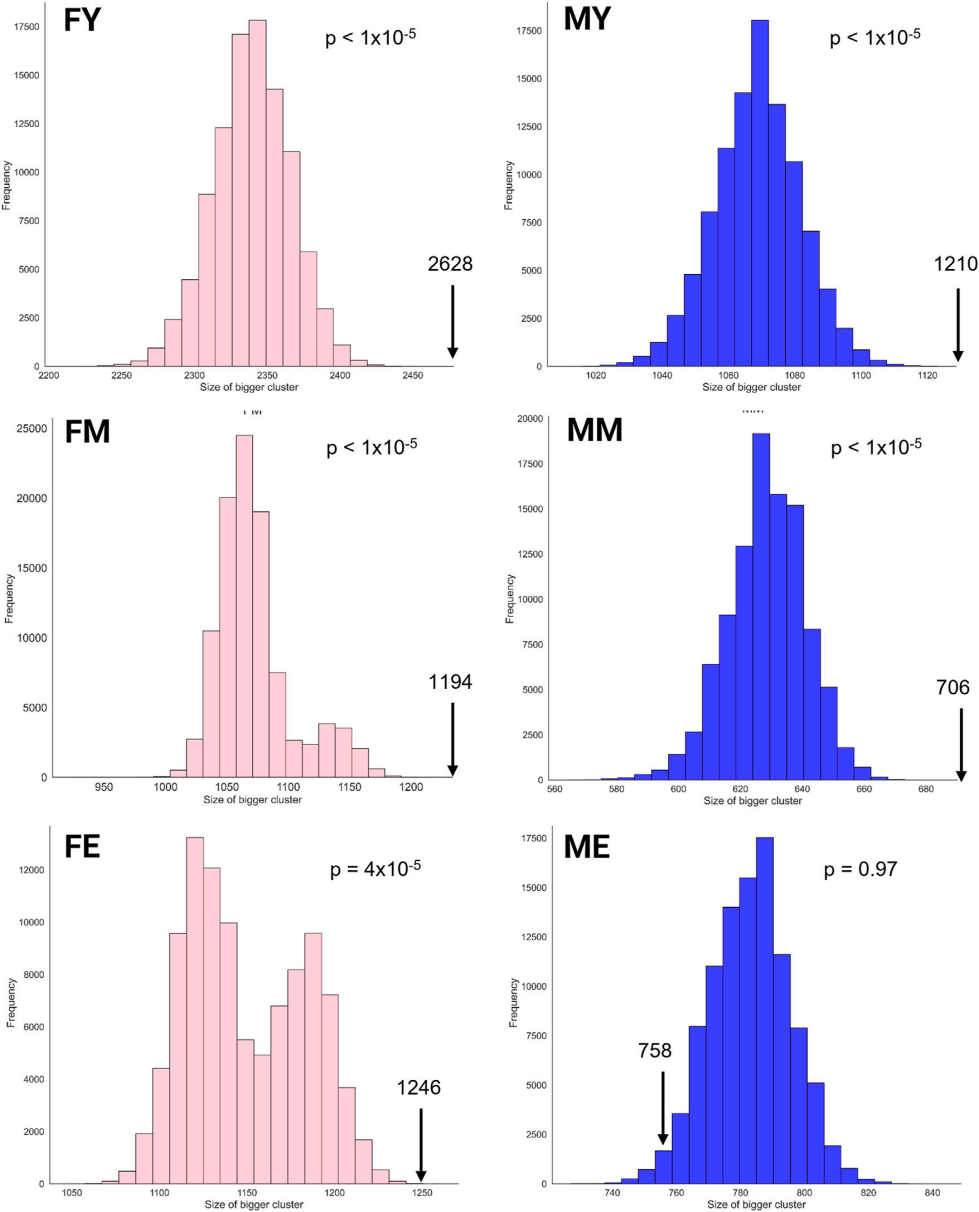
Statistical significance of the largest cluster size in the unique interaction networks.

The unique interaction networks shown in Figure 5 suggest that each sex and age group has its own transcriptional program, at least in the giant component of each network. To investigate this, we questioned whether the size (number of genes) of the largest cluster was not due to chance. Therefore, we developed a null model to test this hypothesis. Figure 6 shows the statistical significance of the largest component size in each network. For nearly all groups, this cluster is larger than expected by chance, reaffirming the hypothesis of unique transcriptional programs for each life stage. However, for ME (elderly men), the cluster is smaller than expected by chance, suggesting that men lose the functional organization of their unique transcriptional program during aging.

**FIGURE 7:**
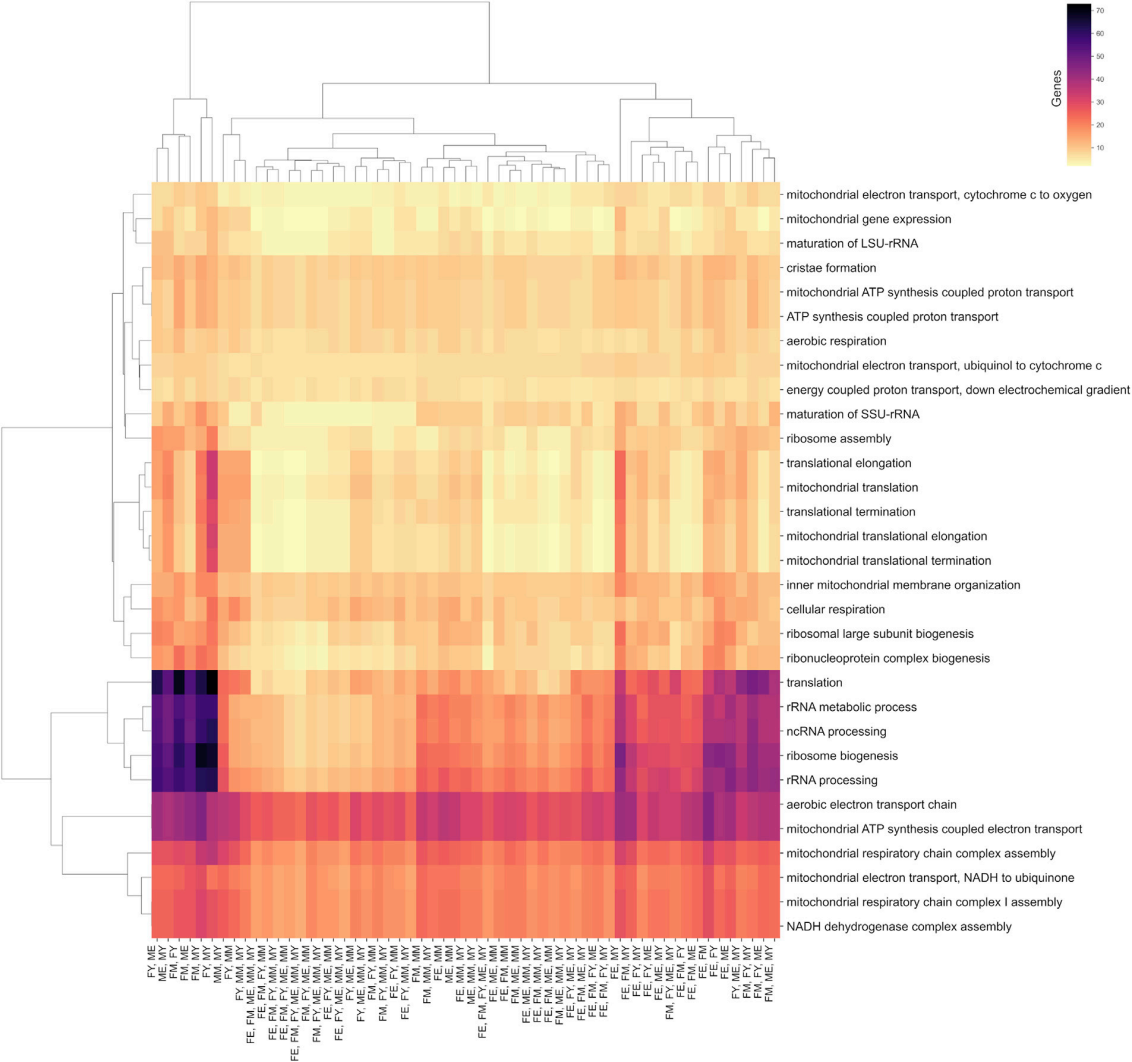
Biological processes shared between at least two age/sex groups. The color scale indicates the number of genes shared in these processes.

Although the different networks have unique interactions, they share common biological processes. Most of these processes are carried out by the same genes, regardless of sex, age, or co-expression patterns. This suggests that some biological processes are so essential for life and muscle maintenance that the mere presence and expression of certain genes can drive the process, irrespective of the functional organization of the genes. Figure 7 shows a clustermap illustrating this, displaying pairs of groups, as well as combinations of groups that, despite not sharing interactions, do share biological processes and genes.

Among the biological processes that best represent this concept are translation, ribosomal biogenesis, and the processing of non-coding and ribosomal RNA. Interestingly, the group that shares the fewest genes in its unique interactions for these processes is FE (elderly women), suggesting that these processes are performed slightly differently in this group.

On the other hand, all groups share almost the same number of genes in mitochondrial processes such as cristae formation, ATP synthesis, electron transport chain, mitochondrial membrane organization, and cellular respiration. This suggests that these essential biological processes are performed by genes that, despite losing or changing their functional coordination, could still carry out the process. However, these changes in coordination might affect the efficiency of the process, potentially explaining the decline in efficiency of these processes during aging.

## Discussion

The loss of muscle mass and functionality associated with aging, known as sarcopenia, is a public health issue as it weakens and disables patients. Diagnosing this complex condition is challenging since there is no official guide for its diagnosis or progression. Sarcopenia exhibits significant sexual dimorphism, with a higher prevalence in men (19.2%) than in women (8.6%) (Du et al., 2019). The reasons for this difference are not yet fully understood, but hormonal changes have been suggested as important factors (Messier et al., 2011).

In this study, we have identified some changes in the functional organization patterns of skeletal muscle genes that exhibit sexual dimorphism and could contribute to the onset and progression of sarcopenia. For instance, women of all ages, unlike men, maintain the functional organization of genes involved in processes such as macroautophagy, sarcomere organization, vesicle coating, protein ubiquitination, and vesicle targeting, which might be essential for muscle maintenance. Moreover, women show a greater number of unique biological processes, suggesting greater flexibility in functional organization. This might mean that the age-related loss of biological processes does not affect women as much, as they possess alternative or complementary processes to the lost ones. Additionally, the unique transcriptional program of each sex/age deteriorates less in women than in men, as elderly women maintain the size of their transcriptional program during aging compared to men.

The biological and molecular mechanisms of sarcopenia are not well understood. However, several have been proposed, including the imbalance between muscle protein synthesis (MPS) and degradation (MPB) (Kim et al., 2020), anabolic resistance (Haran et al., 2012), chronic inflammation (Dalle et al., 2017), poor absorption and metabolism of nutrients, especially amino acids (Cholewa et al., 2017), loss and remodeling of motor units (Kung et al., 2014), ribosomal biogenesis malfunction (Melouane et al., 2019), and mitochondrial dynamics alterations (Melouane et al., 2019). Specifically, we found significant changes in the latter two biological processes, as shown in Figure 7. Our results suggest that changes in co-expression patterns might be responsible for the decline or malfunction of processes associated with mitochondrial dynamics, including mitochondrial gene expression control, mitochondrial membrane organization, electron transport, and ATP production. Interestingly, there is no loss of co-expression of these genes, but significant rewiring of their networks. This rewiring could be a major cause of the decline in mitochondrial functionality associated with aging. Similarly, the functional organization of genes controlling ribosome biogenesis and assembly changes or is lost with age, potentially impacting muscle protein synthesis.

Regarding the common genes in the giant component, as shown in Figure 4 and Supplementary Material S3, two key observations emerge: the number of shared genes is substantial in both cases, and for young phenotypes, there are several unique genes present in the giant component. This finding aligns with the previously mentioned results, which indicate a greater number of biological functions in young phenotypes, particularly concerning reproductive functions in young females. Additionally, the high number of unique genes in young males may suggest an important reservoir of genes that could be activated under certain conditions, with this reservoir diminishing as aging progresses.

The presence of common processes, despite unique gene interactions in each network, exemplifies how processes carrying out essential functions for the organism might adapt to each age’s conditions. Metabolic processes, translation, or ribosomal RNA processing involve more genes in young groups, both men and women, but also in middle-aged and old women. This may be because, during youth, women require a greater number of genes for specific functions, likely related to reproductive, metabolic and hormonal needs. This result of more robust GCNs in females has been previously reported, finding that within the gene modules of female networks there is a greater number of genes compared to males (Sutherland et al., 2019) in addition to occurring in the GCNs of practically all tissues, with these differences in wiring being mainly driven by estrogen (Chen et al., 2016). It has been proposed that these differences could be due to hormonal factors or the effect of sex chromosomes (Van Nas et al., 2009).

Young and elderly groups are expected to differ more in gene expression and biological processes. As illustrated in Figures 3, 4, 7, the number of shared processes between young and elderly phenotypes is lower compared to other age group comparisons. This is largely because networks representing older phenotypes show fewer enriched processes. We hypothesize that this reduction is linked to the loss of genetic plasticity observed with aging, which aligns with both our findings and the current understanding of the aging process. Young individuals usually have more active regenerative capacities, while the elderly might experience more cellular damage accumulation and aging-related changes (Muñoz-Cánoves et al., 2020). This is observed in men, as there are no shared biological processes between young and old men, except for *positive regulation of RNA metabolic process*. However, this prediction does not hold for women, as certain biological processes related to vesicle formation and transport (like *COPII vesicle coating*, *vesicle coating*, and *vesicle targeting, rough ER to cis-Golgi*) are shared between young and old women. These are essential processes for muscle maintenance and function, as well as oxidative stress regulation.

Contrary to expectations, there are few similarities in biological processes between sexes of the same age. Nevertheless, young men and women share some processes, mainly related to protein generation and processing (*mRNA splicing, via spliceosome, positive regulation of transcription initiation from RNA polymerase II promoter, transcription by RNA polymerase I, maturation of 5.8S rRNA, protein-containing complex assembly ERAD pathway, positive regulation of cellular amide metabolic process, actomyosin structure organization*). These processes are related to muscle growth and repair, as young individuals tend to have greater tissue regeneration capacity (Muñoz-Cánoves et al., 2020).

Various studies have reported the loss of gene correlation as a common characteristic of aging (Southworth et al., 2009b; Bernard et al., 2022; Levy et al., 2020). However, our results suggest that the loss of co-expression could begin during middle age, and the changes observed in aging would be a consequence of alterations at earlier ages. Additionally, our results suggest that during aging, gene coordination could be slightly recovered.

The observation that the degradation of gene co-expression networks is more pronounced during middle age, followed by a slight recovery in aging, may be related to several biological and adaptive factors, such as stress response strategies. During middle age, the body experiences significant changes in terms of metabolism, cellular function, and accumulation of molecular damage. This could cause a more abrupt dysregulation in gene coordination. However, in old age, compensatory mechanisms could be activated, such as the activation of cellular stress response pathways (autophagy, chaperones, etc.), which attempt to restore a certain level of homeostasis. This could explain a slight recovery of co-expression in old age. On the other hand, as people age, the most damaged or dysfunctional cells could be eliminated, while those that survive tend to be more resistant. In older people, co-expression networks may be more aligned with cells adapted for survival under chronic stress conditions, which could reflect a partial recovery. Additionally, the middle-age stage may be marked by a transition where cells re-evaluate the balance between growth, repair, and maintenance. As the body ages, there may be a shift towards prioritizing maintenance and survival functions rather than growth, which could lead to a partial stabilization of co-expression networks.

Notably, elderly men do not share any biological processes with elderly women. Elderly men have processes related to enzymatic activity regulation, macromolecule synthesis, and glucose metabolism, suggesting that metabolic processes might be less efficient in elderly men due to changes in co-expression. On the other hand, elderly women exhibit processes related to stress response, post-translational protein modifications, and membrane protein regulation.

It has been proposed that the functioning of a living organism heavily depends on the functional coordination of its genes and that the loss of this coordination leads to the onset and progression of pathological states (Ge et al., 2022). Additionally, it is known that aging leads to a loss of functional organization and gene modularity (Southworth et al., 2009a). However, our results suggest that the loss of functional organization and modularity might begin in middle age (40–59 years), indicating that this loss could be a primary cause of aging-associated deterioration. Thus, we propose the loss of co-expression as a potential new universal marker of aging. This presents an opportunity for developing therapeutic interventions aimed at maintaining or preventing the loss of functional gene coordination, potentially resulting in healthy aging.

## Final considerations and future work

In this study, through a co-expression network analysis, we have been able to identify sets of common and unique processes across three age ranges: youth, maturity, and old age. We also observed these differences by sex. In summary, the main findings of this work can be enumerated as follows.

• Networks of any age and sex present a giant component, suggesting a unique and organized transcriptional program across each phenotype.

• There are many unique interactions for each phenotype, indicating the adaptability of biological processes to each life period.

• Despite unique interactions, the genes that compose each network participate in similar processes. This suggests functional maintenance despite structural modifications, as well as flexibility in developing necessary functions using the available elements in each age range.

• Young and mature women exhibit a greater number of unique processes, likely due to reproductive factors.

• Increasing age does not necessarily imply a loss of functions but rather a decrease in activity.

There are several directions for this work that could help deepen our understanding and the implications of changes and loss in gene functional organization during aging. For example, as observed in our results, investigation on possible co-expression changes in crucial aging-related processes such as macroautophagy and stress response are appealing.

Future work will address the analysis of gene communities, as well as whole-genome-based over-representation analyses, such as GeneSet Enrichment Analysis (Subramanian et al., 2005), the use of deconvolution algorithms to analyze cell composition and proportion and their contribution to each phenotype, using single-cell data to analyze changes in trajectories throughout life, and finally, analyzing the co-expression networks of elderly individuals who have undergone interventions (pharmacological, dietary, or exercise) to identify those interventions that could prevent the loss of gene co-expression.

## Data Availability

The datasets presented in this study can be found in online repositories. The Python scripts used in this study are available in the following repository: https://github.com/Olascoaga/CoexpModularity.

## Supplementary material

SUPPLEMENTARY MATERIAL S1 Networks for the 6 phenotypes under study.

SUPPLEMENTARY MATERIAL S2 List of genes that are loss n the transition form young to middle ages for amle, femal, and both phenotypes. This file also contains the biological processes that dissapear from young to middle ages in the aforementioned cases.

SUPPLEMENTARY MATERIAL S3 Percentage of sharing genes in the giant component of the three different ages for each sex. These Venn diagrams show the number of unique and shared genes for each age for **(A)** females and **(B)** Males.

SUPPLEMENTARY MATERIAL S4 Enriched biological processes for each phenotype under study.
